# Cognitive decline and auditory biomarkers: P300 as a diagnostic tool

**DOI:** 10.1002/alz70856_106478

**Published:** 2026-01-09

**Authors:** Michele da Rocha Anselmo, Lucélia Epifânio Pereira da Silva, Bianca Melo Gonçalves, Lara Catarine Inácio da Silva, Giovanna Correia Pereira Moro, Bruna Fulgêncio Dias, Gabriela Tomé Oliveira Engelmann, Bernardo de Mattos Viana, Maria Aparecida Camargos Bicalho, Ludimila Labanca

**Affiliations:** ^1^ Universidade Federal de Minas Gerais, Belo Horizonte, Minas Gerais, Brazil; ^2^ Cog‐Aging Research Group, Belo Horizonte, Minas Gerais, Brazil; ^3^ Postgraduate Program in Speech‐Language Pathology Sciences, Belo Horizonte, Belo Horizonte, Brazil; ^4^ Postgraduate Program in Speech‐Language Pathology Sciences, Belo Horizonte, Minas Gerais, Brazil; ^5^ Cog‐Aging Research Group, Universidade Federal de Minas Gerais (UFMG), Belo Horizonte, Minas Gerais, Brazil; ^6^ Older Adult's Psychiatry and Psychology Extension Program (PROEPSI), School of Medicine, Universidade Federal de Minas Gerais (UFMG), Belo Horizonte, Minas Gerais, Brazil; ^7^ Sciences Applied to Adult Health Postgraduate Program, School of Medicine, Universidade Federal de Minas Gerais (UFMG), Belo Horizonte, Minas Gerais, Brazil; ^8^ Universidade Federal de Minas Gerais, Belo Horizonte, Brazil; ^9^ Molecular Medicine Program, School of Medicine, Federal University of Minas Gerais, Belo Horizonte, Minas Gerais, Brazil; ^10^ Molecular Medicine Postgraduate Program, School of Medicine, Universidade Federal de Minas Gerais (UFMG), Belo Horizonte, Minas Gerais, Brazil; ^11^ Federal University of Minas Gerais, Belo Horizonte, Minas Gerais, Brazil; ^12^ Department of Clinical Medicine, Faculty of Medicine, Universidade Federal de Minas Gerais (UFMG), Belo Horizonte, Minas Gerais, Brazil; ^13^ Hospital das Clínicas da UFMG, University Hospital, Universidade Federal de Minas Gerais (UFMG), Belo Horizonte, Minas Gerais, Brazil; ^14^ Geriatrics and Gerontology Center Clinical Hospital of Universidade Federal de Minas Gerais, Belo Horizonte, Minas Gerais, Brazil

## Abstract

**Background:**

Over 54% of dementia cases in South America are linked to modifiable factors, emphasizing the need for interventions to prevent or slow its progression. Auditory evoked potentials, electrical signals generated by the auditory system in response to sound stimuli, may serve as valuable diagnostic tools.

**Methods:**

This comparative cross‐sectional study, approved by the institutional ethics committee, included clinical, neuropsychological, and audiological assessments, along with the P300 exam. Participants with severe or profound hearing loss, abnormal tympanometry, traumatic brain injury, stroke, or inability to complete the tests were excluded. A trained audiologist conducted the P300 exam using electrodes placed at the frontal, vertex, and parietal midline, referenced to the ears. Tone bursts of 1000 Hz (frequent) and 2000 Hz (rare) were delivered at 90 dB (300 total stimuli), and participants silently counted rare stimuli. Latencies (N100, P160, N200, P300) and amplitudes (N200‐P300, N100‐P160) were measured by trained examiners. P300 was identified as the highest positive peak (250‐500ms) preceded by N100, P160, and N200. Amplitudes were calculated as peak differences between specific waves.

**Results:**

Fifty‐two individuals participated: 22 with mild cognitive impairment (MCI) and 30 with dementia. In the MCI group, 59% were male (*n* = 13), compared to 27% in the dementia group (*n* = 8) (*p* = 0.024). The mean age was 79 years (SD=4) in the MCI group and 80 years (SD=8) in the dementia group (*p* = 0.274). Educational levels averaged 7 years (SD=5) for MCI and 5 years (SD=4) for dementia (*p* = 0.15). P300 latency was significantly longer in the dementia group (395.63ms [SD=72.11]) compared to the MCI group (355.07ms [SD=53.06]) (*p* = 0.034). No significant differences were observed in N100 (*p* = 0.710), P160 (*p* = 0.860), or N200 (*p* = 0.396) latencies or in amplitudes N200‐P300 (*p* = 0.134) and N100‐P160 (*p* = 0.748).

**Conclusion:**

Dementia patients exhibit prolonged P300 latencies compared to individuals with MCI. These findings highlight the potential of P300 as a biomarker for distinguishing cognitive impairment severity.

